# Health Communication in COVID-19 Era: Experiences from the Italian VaccinarSì Network Websites

**DOI:** 10.3390/ijerph18115642

**Published:** 2021-05-25

**Authors:** Antonella Arghittu, Marco Dettori, Emma Dempsey, Giovanna Deiana, Claudio Angelini, Angela Bechini, Caterina Bertoni, Sara Boccalini, Paolo Bonanni, Sandro Cinquetti, Fabrizio Chiesi, Maria Chironna, Claudio Costantino, Antonio Ferro, Daniel Fiacchini, Giancarlo Icardi, Andrea Poscia, Francesca Russo, Andrea Siddu, Antonietta Spadea, Laura Sticchi, Maria Triassi, Francesco Vitale, Paolo Castiglia

**Affiliations:** 1 Department of Biomedical Sciences, University of Sassari, 07100 Sassari, Italy; arghittu.antonella@gmail.com (A.A.); giovanna.deiana90@gmail.com (G.D.); 2 University Hospital of Sassari, 07100 Sassari, Italy; dempseyemma@gmail.com (E.D.); castigli@uniss.it (P.C.); 3 Department of Medical, Surgical and Experimental Sciences, University of Sassari, 07100 Sassari, Italy; 4 Regional Health Unit Marche, Prevention Department, Hygiene and Public Health Service, 60044 Fabriano, Italy; claudio.angelini@sanita.marche.it (C.A.); daniel.fiacchini@gmail.com (D.F.); 5 Department of Health Sciences, University of Florence, 50134 Florence, Italy; angela.bechini@unifi.it (A.B.); sara.boccalini@unifi.it (S.B.); paolo.bonanni@unifi.it (P.B.); fabrizio.chiesi@unifi.it (F.C.); 6 Department of Prevention, Local Health Authority of Trento, Autonomous Province of Trento, 38123 Trento, Italy; bertonicaterina89@gmail.com (C.B.); antonio.ferro@apss.tn.it (A.F.); 7 Department of Prevention, Local Health Authority 1 Dolomiti, 32100 Belluno, Italy; sandro.cinquetti@aulss1.veneto.it; 8 Dipartimento di Scienze Biomediche e Oncologia Umana, Università degli Studi di Bari, 70124 Bari, Italy; maria.chironna@uniba.it; 9 Department of Health Promotion, Maternal and Infant Care, Internal Medicine and Medical Specialties “G. D’Alessandro”, Section of Hygiene, University of Palermo, 90127 Palermo, Italy; claudio.costantino01@unipa.it (C.C.); francesco.vitale@unipa.it (F.V.); 10 Department of Health Sciences, University of Genoa, 16100 Genoa, Italy; icardi@unige.it (G.I.); sticchi@unige.it (L.S.); 11 UOC ISP Prevention and Surveillance of Infectious and Chronic Diseases, Department of Prevention, Local Health Authority (ASUR-AV2), 60035 Jesi, Italy; andrea.poscia@sanita.marche.it; 12 Regional Directorate of Prevention, Food Safety, Veterinary Public Health, Regione del Veneto, 30123 Venice, Italy; francesca.russo@regione.veneto.it; 13 Ministero della Salute, Ufficio V Prevenzione delle Malattie Trasmissibili e Profilassi Internazionale, 00144 Roma, Italy; a.siddu@sanita.it; 14 UOC Vaccinations, Department of Prevention, Local Health Authority Roma1, 00135 Rome, Italy; antonietta.spadea@aslroma1.it; 15 Department of Public Health, University of Naples “Federico II”, 80131 Naples, Italy; triassi@unina.it

**Keywords:** health communication, e-health, vaccine hesitancy, website, VaccinarSì network

## Abstract

In 2013, in a bid to combat Vaccine Hesitancy (VH) and provide information on vaccines by communicating with the general public and the health community (e.g., healthcare workers and public health operators), the Italian Society of Hygiene and Preventive Medicine (S.It.I.) published the national website “VaccinarSì”. The project was subsequently extended to ten Italian Regions. This led to the creation of the VaccinarSì Network, whose websites are publicly owned. The aim of this work was to present the framework of the websites of the VaccinarSì Network and to analyse user behaviour in the pre-COVID-19-era (dating from each website’s publication until 31 January 2020) and in the COVID-19-era (from 1 February 2020 to 31 January 2021). Some metrics such as the number of visits to the site (sessions, number of users and average session duration), user behaviour (pages viewed, bounce rate and organic search) and the session acquisition path (direct traffic, referrals and social traffic) were searched, extrapolated and processed with Google Analytics. Qualitative and normally distributed quantitative variables were summarised with their absolute (relative) frequencies and means. Statistical differences between the means of the two periods were evaluated through paired *t*-test. A two-tailed *p*-value less than 0.05 was considered to be statistically significant. When the total values recorded over the period were compared, an overall increase in metrics was observed—the number of individual users, visits and individual pageviews rose in a statistically significant way. Our study aimed to highlight how combining disciplines such as health education and digital communication via Information and Communication Technologies (ICT) represents the best strategy to support citizens. This approach gives them the tools to become independent and responsible players that are capable of voluntarily and consciously choosing to adhere to vaccination programs. The VaccinarSì Network’s goal for the future is to reach an even wider audience. By building each user’s critical knowledge, this network enables users to be active components of a wider, more empowered community.

## 1. Introduction

Quick and cheap access to the internet as well as the vast number of registered users of mobile applications (apps) have enabled the Web to become an effective tool for spreading information [1,2,3,4].

Approximately half of the world’s population regularly use social media—5.19 billion people own a mobile phone and 4.54 billion people are regularly connected to the Internet [5]. Moreover, the current pandemic, and the resulting social isolation imposed on the population, has further contributed to the increase in use of digital platforms [6,7,8,9].

For some time now, Public Health has also been making use of modern Information and Communication Technologies (ICT) to reach various population groups and to obtain better health conditions for all [10,11,12]. This practice, known as digital health or e-health, provides healthcare through the use of digital tools (e.g., web sites and social media networks) to be delivered in easy-to-understand language [13,14,15]. On the other hand, digital communication tools in public health, if misused, can act as a conduit for conflicting and misleading information, negatively impacting the health choices of each individual [16,17,18]. In particular, during the COVID-19 pandemic, repeated media exposure to the news, discrepancies in the number of infections and mortality figures reported in different countries, as well as conflicting opinions in the media about the urgent need for a vaccine made it difficult for people to find reliable sources of information [19,20].

Moreover, a low accessibility to technology due to various societal and social factors (i.e., digital divide) make finding correct information even more difficult for those who need it most [21].

Such factors further highlighted the importance of e-health in providing information support for users [22,23,24].

A person’s decision-making process regarding health choices is, indeed, strongly influenced by what they read online, especially in relation to health issues such as vaccination [25,26,27]. According to the World Health Organization (WHO), pandemic-related interruptions in vaccination services and conflicting information on the Internet about SARS-CoV-2 vaccines have further affected vaccination compliance [28,29,30]. In particular, a worrying decline in vaccination coverage for DTaP, measles and polio has been observed in at least 68 countries worldwide, since early 2020 [28,29]. In Italy, during the same period, several regions reported a decrease in childhood and adolescent vaccination coverage for reasons such as—(i) the lack of personnel normally employed in vaccination services, (ii) citizens’ concerns about accessing health services and (iii) unjustified interruptions to active calls for vaccination sessions [24,29].

In this context, the use of e-health by health institutions to counter the phenomenon of Vaccine Hesitancy and to facilitate access to services has acted as an opportunity to educate citizens towards informed and conscious health decisions [31,32,33,34].

Based on the above premises, taking the metrics of the Italian VaccinarSì network (medical and scientific communication websites on vaccinations in Italy) as a starting point, this study aimed to describe and analyse user behaviour recorded before and during the COVID-19 period. It also evaluated the strategies necessary to counteract cyber disinformation through the potential offered by ICTs.

## 2. Materials and Methods

The VaccinarSì website (www.vaccinarsi.org) came into being on 8 May 2013, as part of a health communication project led by the Italian Society of Hygiene (S.It.I.). Its organisational structure consists of three coordinating units—(i) a directive unit (restricted board) composed of 5 experts in Hygiene and Preventive Medicine; (ii) a scientific committee composed of 20 public health professionals whose expertise guarantees the quality of the information published; and (iii) an operational board composed of 36 experts (hygienists, infectivologists, paediatricians and health communication professionals), whose task it is to update information and provide advice/correspondence to users.

In Italy, the reform of Title V of the Constitution (Constitutional Law no. 3 of 18 October 2001) [35], determined the almost exclusive responsibility of the Regions, for the organisation and management of the health service. Meanwhile, the State is responsible for establishing the “essential” health services (Livelli essenziali di assistenza – LEA)—including vaccines—that all Regions must guarantee to citizens, wherever they reside. As such, the Regions, while guaranteeing the vaccines included in the LEAs, are also free to offer further vaccinations to their citizens. From an organisational point of view, they can also use different supply terms and conditions. In the Italian healthcare panorama, therefore, vaccination policies have been characterised by strong territorial heterogeneity. Thus, vaccination information/communication strategies have also needed to be developed at a regional level.

For this reason, in 2014, the Centre for Disease Prevention and Control (CCM) of the Ministry of Health funded a multicentre project that provided for the creation of a VaccinarSì website (VaccinarSì Network) [36] for each of the participating Italian regions (Tuscany: www.vaccinarsintoscana.org; Apulia: www.vaccinarsinpuglia.org; Liguria: www.vaccinarsinliguria.org; Sicily: www.vaccinarsinsicilia.org; Veneto: www.vaccinarsinveneto.org, Latium (www.vaccinarsinlazio.org) and Sardinia: www.vaccinarsinsardegna.org). In the following years, other regions also joined the project: Campania (www.vaccinarsincampania.org), Marche (www.vaccinarsinellemarche.org) and Trentino Alto Adige (www.vaccinarsintrentino.org). In January 2020, the region of Sardinia also published an English-language version of the portal (www.vaccinarsinsardegna.org/en).

The network’s web portals are structurally identical to the national site in terms of organisation and content, apart from one section that is adapted to the territorial context and is specific to each region. The network is hosted on a dedicated server.

### 2.1. The Website Sections

To create the website, a preliminary analysis of the epidemiological framework on the national and international scene was carried out to discern the potential determinants of vaccination hesitation and to develop in-depth information. The contents of the website are organised into 7 sections:

➢ Vaccine-preventable diseases (VPDs): This section contains a brief description of the infectious diseases for which vaccination is available.
➢ Available vaccines: The methods, rules and indications relating to the administration of a vaccine are dealt with in this section. It also contains a direct reference to the corresponding disease.
➢ Vaccine benefits and risks: In this section, the benefits of vaccination and its future prospects are presented and discussed, alongside insights into the real risks related to vaccination practices.
➢ Countering misinformation: The articles in this section analyse and respond to the main theories of the anti-vaccination movements. These movements operate mainly on the Internet, spreading experimental evidence and news that is not supported by data. This results in misleading consequences, inaccurate results, illusory myths and urban legends—or in a word, disinformation.
➢ Travel and vaccinations: In this section of the site, the main issues linking international travel and planned vaccinations are discussed.
➢ News Section: A communication task force is responsible for periodically publishing short news reports on the following topics—epidemiological data on vaccine-preventable infectious diseases (outbreaks and epidemics); immunisation guidelines and programmes; and health education and health communication. Once published on the portal, each news item is automatically forwarded by email to all VaccinarSì subscribers, via a newsletter.
➢ Regional Section: This section provides up-to-date information on vaccination clinics (locations, addresses, opening hours and contacts) in the region, the vaccines offered and terms and conditions, as well as the health promotion initiatives implemented locally.

In order to respond to the various information needs of users, the collection and drafting of the contents present on the VaccinarSì Network is organised into three information levels for each topic covered—(i) title and abstract; (ii) short essay; and (iii) long article.

The network’s activities are described from each portal’s publication date up until 31 January 2021. The network was analysed by distinguishing between a pre-COVID-19 era (from the date of publication to 31 January 2020) and a COVID-19 era (from 1 February 2020 to 31 January 2021). The decision to limit the pre-COVID-19 era to the month of January 2020 relates to the identification of the first European cases of COVID-19, dating back to that period.

The data were searched, extrapolated and processed with Google Analytics, which, using adaptive formulas on the data captured by the software, enabled the analysis of metrics such as: the number of visits to the site (sessions, number of users, average session duration), user behaviour (pages viewed, bounce rate, organic search) and the session acquisition path (direct traffic, referrals and social traffic). The description of the macro-categories and metrics considered in the analysis (glossary) is shown in Table 1.

### 2.2. Statistical Analysis

An ad-hoc electronic form was drawn up to collect the main environmental variables using Excel (Microsoft Office, Microsoft Corporation, Redmond, WA, USA). Qualitative and normally distributed quantitative variables were summarised with absolute (relative) frequencies and means. The statistical differences of means between the two periods were evaluated applying the paired *t*-test. A two-tailed *p*-value less than 0.05 was considered to be statistically significant. The statistical computations were performed using STATA 16 (StatCorp., Austin, TX, USA).

## 3. Results

### 3.1. VaccinarSì Website Activities

The VaccinarSi website was first published on 8 May 2013 as part of a health communication project curated by S.It.I.

It has received the institutional patronage of the Italian Ministry of Health; Istituto Superiore di Sanità-ISS; more than 70 hospital health units and Italian scientific societies involved in immunisation programmes and policies (i.e., Federazione Italiana dei Medici Pediatri—FIMP; Società Italiana di Pediatria—SIP; Federazione Italiana dei Medici di Medicina Generale—FIMMG and the Board of the Calendario Vaccinale per la Vita). In addition, it has been certified by the Health On the Net Foundation (HON), a non-governmental organisation accredited to the United Nations Economic and Social Council and WHO. 

During the study period (8 May 2013–31 January 2021), almost 4.4 million users accessed the portal and almost 18% of these visited the site more than once. In total, the site pages received more than 8.5 million views, with an average session duration of 50 s (Table 1).

In socio-demographic terms, most users came from Italy (95.98%). The site was accessed from abroad—most users came from Switzerland (0.69%), the UK (0.49%), Germany (0.48%) the USA (0.41%) and (1.95%) from 110 other countries. In Italy, the highest number of reported visits to the portal came from Rome (18.5%), Milan (18.2), Naples (3.8%), Turin (3.3%) and Florence (3.1%). Mobile phones were the most commonly used device to access the portal (62.7%), while personal computers (PCs) and tablets accounted for 30.9% and 6.4%, respectively.

Most visitors (80.8%) accessed the site via an Organic search, 13.6% via Direct, 3.6% via Referral and 2.5% through social networks (mainly Facebook) (Table 1). As far as visits to specific web pages are concerned, the most visited pages were those regarding the Immunisation schedule and those dealing with the most common fake news about vaccines (approximately 2500 visits each).

**Figure 1:**
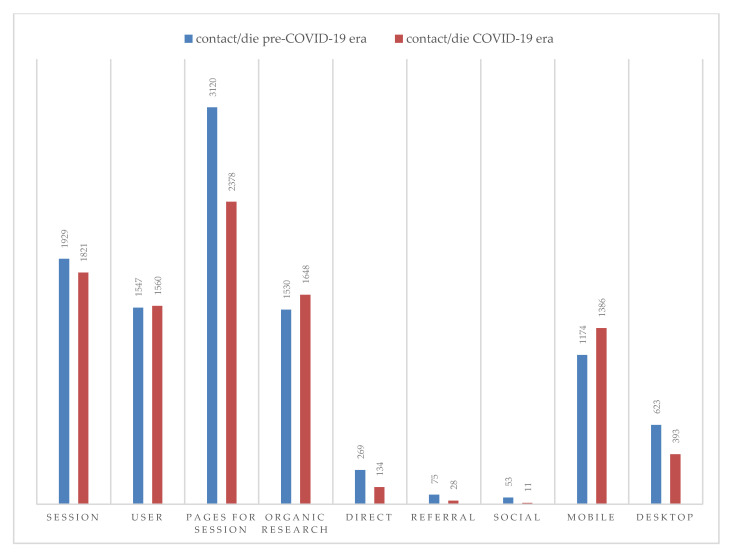
Metrics recorded by the VaccinarSì website per single day during pre-COVID-19 era vs. COVID-19 era.

The metrics recorded by the VaccinarSì website per single day, over the pre-COVID-19 era vs. COVID-19 era (366 days) are shown in Figure 1. 

A general decrease in the number of users was observed during the period under consideration. With regards visits to the website, the number of sessions decreased by 6%, whereas the number of users remained almost unchanged. 

Regarding the behaviour of users during the same period, there was an increase of 8% recorded for organic traffic while web page visits decreased by 24%. This reduction was particularly noticeable when considering the metrics relating to the acquisition of direct, referral and social sessions, for which the number of visits per day fell by 50%, 63% and 80%, respectively.

With regards the devices used to access the site, as compared to the pre-COVID-19 period, during the COVID-19 period, there was an increase of 18% in the use of mobile phones/day (1386 visits/day in the COVID-19 period vs. 1174 visits/day pre-COVID-19) and a reduction of 37% in the use of Personal Computers (393 visits/day in the COVID-19 era vs. 623 accesses/day pre-COVID-19).

### 3.2. VaccinarSì Network Activities

The metrics for the websites of the VaccinarSì network in the period dating from each portal’s publication until 31 January 2021 are reported in Table 2. Similarly, for each network’s web portals, the metrics recorded in the pre-COVID period (from the publication date until 31 January 2020), in the COVID-19 period (from 1 February 2020 to 31 January 2021) and the total are indicated.

With regards to the VaccinarSì Network, a comparison of the total values recorded in the pre-COVID-19 era and during the COVID-19 era showed an overall increase in the following metrics—(i) bounce rate (81.58% in COVID-19 period vs. 79.79% pre-COVID-19; (ii) organic search (88.85% in COVID-19 period vs. 79.80% pre-COVID-19); and (iii) mobile device usage (73.71% in COVID-19 period vs. 61.50% pre-COVID-19). On the other hand, a decrease in the recorded values was observed in relation to—(iv) direct, referral and social acquisition paths and (v) PC and tablet use.

In particular, the increase of the organic search path, to the detriment of the referral and social paths, was observed for 9 of the 12 websites.

**Figure 2:**
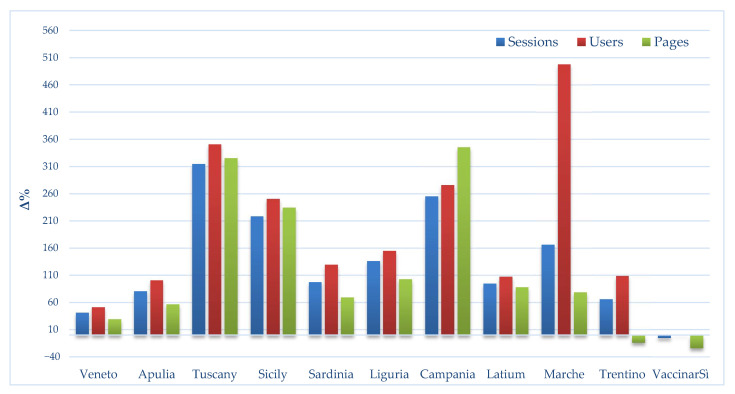
Percentage difference in absolute values of users, sessions and pageviews on network websites in the pre-COVID-19 and COVID-19 eras.

The differences in session, user and pageview metrics in the COVID-19 period as compared to the pre-COVID-19 period are shown in Figure 2.

From Figure 2 it can be seen that all regional sites recorded an increase in the metrics in question (range between +41.5% and +498%), with the highest values for the number of users (from +51.6% to +498.1%) with an exception for the number of pageviews on the vaccinarsintrentino.org website, which showed a decrease of 13.8% in the COVID-19 period.

Table 3 shows the statistical differences found in the number of sections, users and pages registered per day, during the pre-COVID-19 and COVID-19 era.

In particular, as far as all the websites of the VaccinarSì network are concerned, the paired *t*-test performed showed a significant statistical difference in the number of sections, users and pages registered during the pre-COVID-19 and COVID-19 eras. 

This increased consultation of the regional websites corresponds to a slight decrease in the number of sessions and pages viewed on the national website.

**Figure 3:**
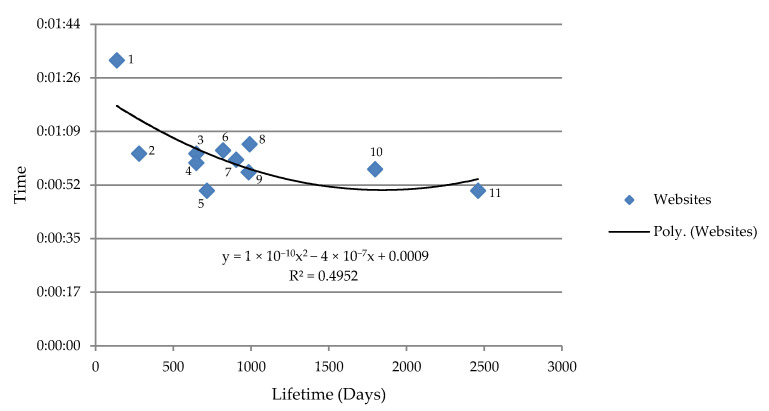
Average session consultation time in relation to the lifespan of each of the websites. (1) www.vaccinarsintrentino.org; (2) www.vaccinarsinellemarche.org; (3) www.vaccinarsincampania.org; (4) www.vaccinarsinlazio.org; (5) www.vaccinarsinliguria.org; (6) www.vaccinrsinsardegna.org; (7) www.vaccinarsinsicilia.org; (8) www.vaccinarsinpuglia.org; (9) www.vaccinarsintoscana.org; (10) www.vaccinarsinveneto.org; and (11) www.vaccinarsi.org.

The average consultation time appears to have decreased over the last year for all websites in the network. The average consultation time of the sessions in relation to the lifespan of each website is shown in Figure 3 (the ordinate shows the consultation time during the era of COVID-19 while the x-axis shows the websites’ lifespan).

The graph shows that the average consultation time decreased in a quadratic manner in accordance with the website’s age. The sites with the shortest lifespan have the longest session duration (e.g., www.vaccinarsintrentino.org with 0:01:32 min of consultation time).

## 4. Discussion

Web-based vaccination content is not always well-regulated and the spread of erroneous and misleading information cannot be limited or monitored [37,38,39]. Many young people and families rely on the information available on the Internet when choosing whether to adhere to vaccination programmes. Using the Internet to inform the population about the importance of vaccinations is a strategic tool for Public Health. As such, the aim of the VaccinarSì project was to provide in-depth online information by strategically exploiting modern ICT and e-Health [32,37]. In these years of activity, the website has promoted the culture of preventive vaccination, constantly expanding the user-base with clicks to the site coming from over 100 countries. The views originating from outside Italy might stem from the website’s inclusion in the 53 web portals of the Vaccine Safety Net network, also increasing its visibility on the international scene [40,41].

From a careful observation of the metrics recorded per day in the pre-COVID-19 and COVID-19 eras, we can see that, while on the one hand, the VaccinarSì website showed a decreasing trend with regards pageviews, on the other hand, the network showed an increase. Indeed, some regional web portals saw their number of visits triple. This phenomenon also reflects the Italian regions’ autonomy of management, with regards to the anti-COVID-19 vaccination plan implemented in Italy.

According to Zhao et al., the isolation imposed during the pandemic led to an increase in the use of digital social platforms to look up information [16,17]. This is also evident from our observations, as for both the national and regional portals, there was an increase in the use of mobile devices as compared to desktop ones. 

As far as user behaviour is concerned, an increase in organic traffic was observed in the period under consideration, to the detriment of the referral and social paths of acquisition. This phenomenon, which was found in 9 out of 13 sites, confirmed the network as a preferred tool for direct search to acquire information on health, mainly on vaccines. The network’s strength is the publication of evidence-based topics, with a particular focus on local and regional information.

Moreover, considering the number of sessions, users and pages recorded by the VaccinarSì network, there were differences regarding daily visits to the site (i.e., sessions and users) and user behaviour (i.e., pages viewed). In the COVID-19 period as compared to the pre-COVID-19 period, we can see statistically significant differences, with an increasing trend for all regional websites, with an exception of the number of pages viewed on vaccinarsintrentino.org as well as for the national site. These data are difficult to interpret in light of the short pre-COVID-19 lifespan of the Trentino regional website and the instability of the statistics calculated. In contrast, for the VaccinarSì website (which has the longest pre-COVID-19 lifespan), a reduction in the number of pages consulted was observed. This may be linked to the fact that users may be more interested in the News items published rather than in the site’s other previously known pages (the Science and Knowledge section, which is common to all of the portal’s websites).

Finally, with regards to the average session duration in relation to each web site’s lifespan, our results show that with newer sites (e.g., vaccinarsintrentino.org) users spent a longer time browsing the portal than they do with older sites (e.g., www.vaccinarsi.org). This could be related to the users’ level of confidence with each individual web site; indeed, a medical and scientific information site on vaccinations known to the population by virtue of its age requires less consultation time than a site with similar contents but more recently published.

## 5. Conclusions

The results of our study might imply an effect of the phenomenon of emotional epidemiology, which affected the entire population in the COVID-19-era. Indeed, in the early stages of a pandemic, the public was keen to seek relevant knowledge and information online to meet their protection needs [42,43]. This phenomenon, together with the social isolation imposed as a measures to contain the spread of SARS-CoV-2, may have led to an exacerbated use of ICT. Thus, the increase in our metrics may also depend on this. Nevertheless, the average time of consultation and the number of pages viewed by users are useful indicators of positive feedback and community loyalty to the VaccinarSì network.

The ICT have become an integral part of our everyday lives and the development of modern ICT has given a further push towards digital consumption [1,2]. Given technology’s potential in information-seeking processes, the use of online channels by healthcare institutions is a valuable tool for the dissemination of medical and scientific knowledge to patients. In this context, recently published protocols [13,14,15,19,20,22,23] and systematic literature reviews describe the importance of e-Health applied to certain healthcare contexts [23,24,25,26,27,28,29,30,31,32,33,34]. In particular, these describe how the use of online channels, instant messaging (SMS) or Short-Messages sent via apps to communicate with patients can—(i) facilitate user participation by increasing the attendance rate for health appointments [13]; (ii) tackle the misinformation prevailing on the web; (iii) improve therapeutic compliance [27,28,29,30,31]; and (iv) provide remote psychological support in patients with particular clinical conditions [31,32]. Conversely, digital communication tools in Public Health can act as a channel for incorrect and misleading information [36,37,38,39].

In line with what has already been described in the literature, the results of the VaccinarSì Network Project highlight, in particular in the current pandemic context, the need to invest in health literacy by making full use of the potential offered by ICT. Our study aimed to highlight how uniting disciplines such as health education and digital communication may well be the best strategy to support citizens, by enabling them to independently, responsibly and consciously choose whether to voluntarily adhere to vaccination programs [16,40].

In fact, the transfer of shared knowledge and skills through institutional websites, with dedicated sections, periodic news and scientific publications made available, aims to build each user’s critical knowledge, thereby making each individual an active part of an extended community (empowered organisation) in continuous growth, and paving the way for increased evidence and experience-based knowledge [17,42]. In our study, the result of the active empowerment process is clear to see, above all from the user-loyalty which serves to measure impact on the community and user satisfaction. In fact, with a 90% increase in the volume of online conversations following requests for information through virtual consultancy help-desks (especially in the COVID-19-era), the services provided by the network have become increasingly well-known, and the project’s impact in terms of improving compliance and citizens’ awareness has grown.

Gaps such as the lack of adequate educational methods in the health sector along with the presence of misleading information content on the web, contribute to altering perceptions of health risk and increasing improper spreading of information, especially with regards to the topic of vaccinations. On the contrary, proper sharing of verified scientific content and health communication via mass media, provides users with the necessary tools to make informed health decisions. At the same time, it gives them the chance to become autonomous in searching and disseminating reliable information [16,17,18,44].

In this context, features such as total views, pageviews, bounce rate and time spent on a specific landing page, are useful cyber information indicators. In our study, these demonstrate the importance of digital vaccine communication initiatives such as a support for users, operators and the community. In this context, a key role is played by the active cooperation between the media and scientific professionals (experts in public health, infectious diseases and epidemiology), who, in collaboration with local doctors (general practitioners and paediatricians) could constitute an effective network in identifying and ‘unmasking’ disinformation, by providing the public with simple and relevant sources of up-to-date information. The future use of these digital tools to disseminate reliable information, represents an opportunity for the transfer of evidence-based knowledge for users and health professionals, in order to achieve the empowerment of the population for informed health choices.

The VaccinarSì Network’s goal for the future is to grow further to reach an even wider audience and take advantage of further collaborations with national and international scientific organisations and associations, to further promote the culture of prevention and vaccination.

## Figures and Tables

**Table 1:** Glossary.

Macrocategory	Metric	Definition
Visits to the site	Session	A single continual active viewing period by a visitor.
	User	The number of people that have visited your site at least once during a given time-period. One user could have multiple sessions, but will still be counted as a single user.
	Average Session Duration in seconds	The Average Session Duration provides a top-level view of how long users spend on your site.
Behaviour	Pageviews	The total number of website pages viewed. For example, if one user visited your homepage and the contact page, then that would count as two pageviews.
	% Bounce rate	The percentage of sessions with a single pageview (a sign of NON-transitability within the website).
Session acquisition	Organic	Traffic originating from natural (or unpaid) results on search engines.
	Direct	Direct traffic includes people who typed your website’s URL into their browser or who had it memorised in their browser.
	Referral	A referral is reported when a user clicks through to your website from another third-party website (not social media).
	Social	Number of visits to the site via a link or reference from social media portals.
Device	Desktop	Sessions occurring on desktop (PC or laptop).
	Mobile	Sessions occurring on mobile phones
	Tablet	Sessions occurring on Tablet o iPad

**Table 2:** Metrics for the VaccinarSì network websites in the pre-COVID-19 era (from publication date to 31 January 2020); during the COVID-19 era (from 1 February 2020 to 31 January 2021) and the total (from publication date of each portal until 31 January 2021).

			Visits to Site			Behaviour		Session Acquisition				Device		
Website	Period	Days (*n*)	Sessions (*n*)	Users (*n*)	Session (s)	Pageviews (*n*)	Bounce (%)	Organic (%)	Direct (%)	Referral (%)	Social (%)	Desktop (%)	Mobile (%)	Tablet (%)
**VaccinarSì**	pre-era	2460	4,744,925	3,805,888	0:00:50	7,675,317	80.14	79.34	13.95	3.9	2.76	32.30	60.85	6.86
	COVID-era	366	666,655	571,017	0:00:50	870,228	87.04	90.94	7.38	1.54	0.6	21.56	76.11	2.34
	tot	2826	5,411,580	4,376,905	0:00:50	8,545,545	80.99	80.77	13.14	3.61	2.49	30.98	62.73	6.27
**VENETO**	pre-era	1798	506,136	433,208	0:01:06	855,215	78.74	84.14	12.54	2.64	0.69	30.98	63.97	5.06
	COVID-era	366	145,832	125,911	0:00:57	225,373	80.15	91.90	6.58	1.32	0.2	25.67	71.64	2.69
	tot	2163	651,968	559,119	0:01:04	1,080,588	79.06	85.88	11.21	2.34	0.58	29.79	65.69	4.53
**APULIA**	pre-era	991	49,066	41,252	0:01:26	96,892	71.72	64.44	17.49	5.23	12.84	32.68	63.6	3.72
	COVID-era	366	32,771	28,744	0:01:05	56,056	72.71	83.87	12.86	2.04	1.17	29.65	68.37	1.98
	tot	1357	81,837	69,996	0:01:17	152,948	72.12	72.22	15.64	3.95	8.17	31.47	65.51	3.01
**TUSCANY**	pre-era	984	58,853	48,880	0:01:03	94,166	79.48	83.66	7.79	5.78	2.77	31.51	65.32	3.17
	COVID-era	366	90,765	77,501	0:00:56	149,141	76.15	84.03	12.66	0.57	2.74	27.5	69.73	2.77
	tot	1350	149,618	126,381	0:00:59	243,307	77.68	83.88	10.74	2.62	2.75	29.08	68.00	2.77
**SICILY**	pre-era	904	45,656	38,537	0:01:12	74,832	77.02	73.01	11.97	4.41	10.6	26.9	69.68	3.42
	COVID-era	366	58,902	51,318	0:01:00	101,313	72.27	78.45	10.81	2.62	8.12	25.07	72.67	2.26
	tot	1270	104,558	89,855	0:01:05	176,145	74.34	76.07	11.32	3.40	9.20	25.87	71.36	2.76
**SARDINIA ITA**	pre-era	820	39,887	32,040	0:01:39	83,243	71.10	74.52	17.62	5.34	2.52	32.93	62.65	4.43
	COVID-era	366	35,178	30,693	0:01:03	63,060	70.98	83.31	11.62	3.62	1.45	31.96	65.26	2.79
	tot	1186	75,065	62,733	0:01:22	146,303	71.04	78.64	14.81	4.53	2.02	32.48	63.87	3.63
**SARDINIA EN**	pre-era	0	0	0	0:00:00	0	0.00	0.00	0	0	0	0	0	0
	COVID-era	366	1613	1446	0:00:49	2929	78.74	74.15	14.76	5.27	5.83	52.39	44.45	3.16
	tot	366	1613	33,486	0:01:37	2929	78.74	74.15	14.76	5.27	5.83	52.39	44.45	3.16
**LIGURIA**	pre-era	715	13,500	11,665	0:00:54	21,567	78.59	84.47	12.9	2.42	0.19	29.54	66.59	3.87
	COVID-era	366	16,316	14,779	0:00:50	24,474	79.27	80.23	9.59	10.14	0.04	23.51	73.45	3.04
	tot	1081	29,816	26,444	0:00:52	46,041	78.96	82.15	11.09	6.64	0.11	26.24	70.34	3.41
**CAMPANIA**	pre-era	649	39,150	32,776	0:01:03	57,485	81.36	91.96	7.38	0.48	0.18	21.63	76.28	2.08
	COVID-era	366	78,380	66,980	0:01:02	144,347	68.90	89.41	8.46	1.65	0.4	22.21	75.76	2.03
	tot	1015	117,530	32,776	0:01:02	201,832	73.05	90.26	8.10	1.26	0.33	22.02	75.93	2.05
**LATIUM**	pre-era	648	65,587	56,433	0:01:09	100,942	77.89	89.32	7.84	1.49	1.34	29.8	66.55	3.65
	COVID-era	366	72,290	63,641	0:00:59	107,283	78.52	87.00	7.74	5.06	0.2	27.94	69.28	2.78
	tot	1014	137,877	120,074	0:01:04	208,225	78.22	88.10	7.79	3.36	0.74	28.82	67.98	3.19
**MARCHE**	pre-era	281	12,754	9708	0:01:15	22,400	75.92	81.34	13.49	2.35	2.81	28.13	70.05	1.82
	COVID-era	366	44,297	35,096	0:01:02	70,790	76.77	87.11	11.25	1.55	0.09	25.43	72.65	1.92
	tot	647	57,051	44,804	0:01:05	93,190	76.58	85.82	11.75	1.73	0.70	26.03	72.07	1.90
**TRENTINO**	pre-era	137	2458	1704	0:03:43	11,784	34.38	31.00	35.27	31.04	2.69	49.51	47.64	2.85
	COVID-era	366	10,900	8767	0:01:32	27,124	55.22	80.26	12.82	6.82	0.11	34.9	61.19	3.91
	tot	503	13,358	10,471	0:01:56	38,908	51.39	71.20	16.95	11.28	0.58	37.59	58.70	3.74
**VaccinarSì Network**	pre-era	10,387	5,577,972	4,512,091	0:53:00	9,093,843	79.79	79.8	13.68	3.78	2.69	32.04	61.5	6.49
	COVID-era	4392	1,253,899	1,075,893	0:55:00	1,842,118	81.58	88.85	8.4	1.93	1.04	23.86	73.71	2.42
	tot	14,779	6,831,871	5,587,984	0:53:00	10,935,961	80.12	81.46	12.71	3.44	2.38	30.53	63.74	5.74

**Table 3:** VaccinarSì Network. Statistical differences in the number of sections, users and pages per day during the pre-COVID-19 and COVID-19 eras.

Website	No. Sections/day		Paired T-Student	No. Users/day		Paired T-Student	No. Pages/day		Paired T-Student
	pre	post	*p*-value	pre	post	*p*-value	pre	post	*p*-value
Veneto	281	398	0.001	227	344	0.0007	475	616	0.003
Apulia	50	90		39	79		98	153	
Tuscany	60	248		47	212		96	407	
Sicily	51	161		40	140		83	277	
Sardinia	49	96		37	84		102	172	
Liguria	19	45		16	40		30	67	
Campania	60	214		49	183		89	394	
Latium	101	198		84	174		156	293	
Marche	45	121		33	96		80	193	
Trentino	18	30		11	24		86	74	

## Data Availability

The data presented in this study are available on reasonable request from the corresponding author.

## References

[1] Kapoor K.K., Tamilmani K., Rana N.P., Patil P.P., Dwivedi Y.K., Nerur S. (2018). Advances in Social Media Research: Past, Present and Future. Inf. Syst. Front..

[2] Soler-Costa R., Lafarga-Ostáriz P., Mauri-Medrano M., Moreno-Guerrero A.J. (2021). Netiquette: Ethic, Education, and Behavior on Internet-A Systematic Literature Review. Int. J. Environ. Res. Public Health.

[3] Vaterlaus J.M., Aylward A., Tarabochia D., Martin J.D. (2021). “A smartphone made my life easier”: An exploratory study on age of adolescent Smartphone acquisition and well-being. Comput. Hum. Behav..

[4] Park C.S., Kaye B.K. (2019). Smartphone and self-extension: Functionally, anthropomorphically, and ontologically extending self via the Smartphone. Mob. Media Commun..

[5] Report Digital 2020 We Are Social e Hootsuite. https://wearesocial.com/it/blog/2020/01/report-digital-2020-i-dati-global.

[6] González-Padilla D.A., Tortolero-Blanco L. (2020). Social media influence in the COVID-19 Pandemic. Int. Braz. J. Urol..

[7] Brooks S.K., Webster R.K., Smith L.E., Woodland L., Wessely S., Greenberg N., Rubin G.J. (2020). The psychological impact of quarantine and how to reduce it: Rapid review of the evidence. Lancet.

[8] Li C., Chen L.J., Chen X., Zhang M., Pang C.P., Chen H. (2020). Retrospective analysis of the possibility of predicting the COVID-19 outbreak from Internet searches and social media data. China, 2020. Eurosurveillance.

[9] Ming L.C., Untong N., Aliudin N.A., Osili N., Kifli N., Tan C.S., Goh K.W., Ng P.W., Al-Worafi Y.M., Lee K.S. (2020). Mobile Health Apps on COVID-19 Launched in the Early Days of the Pandemic: Content Analysis and Review. JMIR mHealth uHealth.

[10] mHealth: Use of Appropriate Digital Technologies for Public Health: Report by the Director-General. World Health Organization, 2017. https://apps.who.int/iris/handle/10665/274134.

[11] Chen Y.R., Schulz P.J. (2016). The Effect of Information Communication Technology Interventions on Reducing Social Isolation in the Elderly: A Systematic Review. J. Med. Internet Res..

[12] Hilty D., Chan S., Torous J., Luo J., Boland R. (2020). A framework for competencies for the use of mobile technologies in psychiatry and medicine: Scoping review. JMIR mHealth uHealth.

[13] Free C., Phillips G., Felix L., Galli L., Patel V., Edwards P. (2010). The effectiveness of M-health technologies for improving health and health services: A systematic review protocol. BMC Res. Notes.

[14] Materia F.T., Faasse K., Smyth J.M. (2020). Understanding and preventing health concerns about emerging mobile health technologies. JMIR mHealth uHealth.

[15] Liao C.Y., Liu W.I. (2020). Current Status and Prospects for Information Communication Technology (ICT) in Allied Health Education. Hu Li Za Zhi.

[16] Zhao Y., Xu H. (2020). Chinese public attention to COVID-19 epidemic: Based on social media. medRxiv.

[17] Banerjee D., Meena K.S. (2021). COVID-19 as an “Infodemic” in Public Health: Critical Role of the Social Media. Front. Public Health.

[18] Gentili D., Bardin A., Ros E., Piovesan C., Ramigni M., Dalmanzio M., Dettori M., Filia A., Cinquetti S. (2020). Impact of Communication Measures Implemented During a School Tuberculosis Outbreak on Risk Perception among Parents and School Staff, Italy, 2019. Int. J. Environ. Res. Public Health.

[19] Dettori M., Deiana G., Balletto G., Borruso G., Murgante B., Arghittu A., Azara A., Castiglia P. (2021). Air pollutants and risk of death due to COVID-19 in Italy. Environ. Res..

[20] Deiana G., Azara A., Dettori M., Delogu F., Vargiu G., Gessa I., Stroscio F., Tidore M., Steri G., Castiglia P. (2020). Deaths in SARS-Cov-2 Positive Patients in Italy: The Influence of Underlying Health Conditions on Lethality. Int. J. Environ. Res. Public Health.

[21] Ramsetty A., Adams C. (2020). Impact of the digital divide in the age of COVID-19. J. Am. Med Inform. Assoc..

[22] O’Connor Y., Andreev P., O’Reilly P. (2020). MHealth and perceived quality of care delivery: A conceptual model and validation. BMC Med. Inform. Decis. Mak..

[23] Gupta L., Gasparyan A.Y., Misra D.P., Agarwal V., Zimba O., Yessirkepov M. (2020). Information and Misinformation on COVID-19: A Cross-Sectional Survey Study. J. Korean Med. Sci..

[24] Zhang L., Li H., Chen K. (2020). Effective Risk Communication for Public Health Emergency: Reflection on the COVID-19 (2019-nCoV) Outbreak in Wuhan, China. Healthcare.

[25] Arghittu A., Dettori M., Azara A., Gentili D., Serra A., Contu B., Castiglia P. (2020). Flu Vaccination Attitudes, Behaviours, and Knowledge among Health Workers. Int. J. Environ. Res. Public Health.

[26] Dettori M., Arru B., Azara A., Piana A., Mariotti G., Camerada M.V., Stefanelli P., Rezza G., Castiglia P. (2018). In the Digital Era, Is Community Outrage a Feasible Proxy Indicator of Emotional Epidemiology? The Case of Meningococcal Disease in Sardinia, Italy. Int. J. Environ. Res. Public Health.

[27] Carducci A., Fiore M., Azara A., Bonaccorsi G., Bortoletto M., Caggiano G., Calamusa A., De Donno A., De Giglio O., Dettori M. (2019). Environment and health: Risk perception and its determinants among Italian university students. Sci. Total Environ..

[28] World Health Organization (2020). Guiding Principles for Immunization Activities during the COVID-19 Pandemic. Interim Guidance.

[29] Bonanni P., Angelillo I.F., Villani A., Biasci P., Scotti S., Russo R., Maio T., Rosati G.V., Barretta M., Bozzola E. (2021). Maintain and increase vaccination coverage in children, adolescents, adults and elderly people: Let’s avoid adding epidemics to the pandemic: Appeal from the Board of the Vaccination Calendar for Life in Italy: Maintain and increase coverage also by re-organizing vaccination services and reassuring the population. Vaccine.

[30] Costantino C., Fiacchini D. (2020). Rationale of the WHO document on Risk Communication and Community Engagement (RCCE) readiness and response to the Severe Acute Respiratory Syndrome Coronavirus 2 (SARS-CoV-2) and of the Italian Decalogue for Prevention Departments. J. Prev. Med. Hyg..

[31] Wiysonge C.S., Ndwandwe D., Ryan J., Jaca A., Batouré O., Anya B.M., Cooper S. (2021). Vaccine hesitancy in the era of COVID-19: Could lessons from the past help in divining the future?. Hum. Vaccin. Immunother..

[32] Wilson S.L., Wiysonge C. (2020). Social media and vaccine hesitancy. BMJ Global Health.

[33] Ferro A., Odone A., Siddu A., Colucci M., Anello P., Longone M., Marcon E., Castiglia P., Bonanni P., Signorelli C. (2015). Monitoring the web to support vaccine coverage: Results of two years of the portal VaccinarSì. Epidemiol. Prev..

[34] Karafillakis E., Dinca I., Apfel F., Cecconi S., Wűrz A., Takacs J., Suk J., Celentano L.P., Kramarz P., Larson H.J. (2016). Vaccine hesitancy among healthcare workers in Europe: A qualitative study. Vaccine.

[35] Presidenza della Repubblica Italiana Legge Costituzionale del 18 ottobre 2001, n. 3 Modifiche al Titolo V della Parte Seconda Della Costituzione. (GU Serie Generale n.248 del 24-10-2001). https://www.gazzettaufficiale.it/eli/id/2001/10/24/001G0430/sg.

[36] Ministero della Salute Centro Nazionale per la Prevenzione e il Controllo delle Malattie. Programma CCM 2014. “Monitorare la Fiducia del Pubblico nei Programmi Vaccinali e le sue Necessità Informative Sviluppando un Sistema di Decisione Assistita per le Vaccinazioni Tramite il Sito “vaccinarsi.org” e Altri Siti e Social Network Specificatamente Dedicati Alle Vaccinazioni”. http://www.ccm-network.it/progetto.jsp?id=node/1884&idP=740.

[37] Kata A. (2012). Anti-vaccine activists, Web 2. 0, and the postmodern paradigm—An overview of tactics and tropes used online by the anti-vaccination movement. Vaccine.

[38] Fiacchini D., Icardi G., Lopalco P.L., Conversano M. (2019). Comunicare i Vaccini per la Salute Pubblica.

[39] Boccalini S., Bonanni P., Chiesi F., Pisa G.D., Furlan F., Giammarco B., Zanella B., Mandò Tacconi F., Bechini A. (2020). The Experience of VaccinarSinToscana Website and the Role of New Media in Promoting Vaccination. Vaccines.

[40] World Health Organization Vaccine Safety Network. https://www.vaccinesafetynet.org/vsn/network/vaccinars%C3%AC.

[41] Costantino C., Caracci F., Brandi M., Bono S.E., Ferro A., Sannasardo C.E., Scarpitta F., Siddu A., Vella C., Ventura G. (2020). Determinants of vaccine hesitancy and effectiveness of vaccination counseling interventions among a sample of the general population in Palermo, Italy. Hum. Vaccin. Immunother..

[42] Gu H., Chen B., Zhu H., Jiang T., Wang X., Chen L., Jiang Z., Zheng D., Jiang J. (2014). Importance of Internet surveillance in public health emergency control and prevention: Evidence from a digital epidemiologic study during avian influenza A H7N9 outbreaks. J. Med. Internet Res..

[43] Chen Y., Zhang Y., Xu Z., Wang X., Lu J., Hu W. (2019). Avian Influenza A (H7N9) and related Internet search query data in China. Sci. Rep..

[44] European Centre for Disease Prevention and Control Technical Document. Social Media Strategy Development. A Guide to Using So Media for Public health Communication. https://www.ecdc.europa.eu/sites/default/files/media/en/publications/Publications/social-media-strategy-guide-for-public-health-communication.pdf.

